# Improving Insights of Medical Students Regarding Sexually Transmitted Diseases: A Quality Improvement Project

**DOI:** 10.7759/cureus.102173

**Published:** 2026-01-23

**Authors:** Sude Cavdaroglu, Atakan Yuksel, Sevval Ilayda Sönmez, David Terence Thomas

**Affiliations:** 1 Emergency Medicine, Arnavutkoy State Hospital, Istanbul, TUR; 2 General Practice, Umraniye District Health Directorate, Istanbul, TUR; 3 Pediatrics and Child Health, Istanbul Zeynep Kamil Maternity and Children’s Diseases Health Training and Research Center, Istanbul, TUR; 4 Pediatric Surgery, Hamidiye Faculty of Medicine, University of Health Sciences, Istanbul, TUR; 5 Pediatric Surgery, Sancaktepe Şehit Prof.Dr. İlhan Varank Training and Research Hospital, Istanbul, TUR

**Keywords:** clinical year medical students, peer education, plan-do-study-act (pdsa), quality improvement project, sexually transmitted diseases (stds)

## Abstract

Introduction: Sexually transmitted diseases (STDs) remain a critical component of medical education, yet gaps in knowledge and misconceptions persist among students due to overwhelming curricula and lack of clinical integration.

Methods: This quality improvement project (QIP), conducted at Maltepe University Faculty of Medicine, aimed to enhance students’ understanding of STDs through a dual-cycle intervention using the plan-do-study-act (PDSA) framework. A baseline survey (n=180) revealed inconsistent knowledge levels and prevalent misconceptions across academic years. In response, two interventions, peer-led educational presentations and informative posters, were implemented based on participant feedback. Knowledge was assessed using 20-item surveys before and after each intervention. To evaluate the effectiveness of the educational interventions, knowledge levels were assessed on a 5-point scale (1=None to 5=Very Good). The dataset was analyzed as independent samples because the student-by-student pairing (ID mapping) was not available in the summary file. A Mann-Whitney U Test and an independent samples t-test were also performed to compare the mean scores, which are common metrics in medical education literature.

Results: The average knowledge score improved significantly from 3.56 to 4.42 following peer education and from 3.71 to 4.28 after poster exposure. Specific knowledge of sexually transmitted infections such as trichomoniasis and hepatitis B increased markedly, while myths surrounding transmission routes were partially corrected. Students rated the presentation more helpful than posters (4.33 vs. 3.79 on a 5-point scale).

Conclusion: Despite limitations including low participation during exam periods, the interventions proved effective and well-received. This project underscores the value of peer-led and visual education in bridging knowledge gaps and student-tailored sexual health education in medical curricula.

## Introduction

During their medical studies, medical students receive a wide range of theoretical information on sexually transmitted diseases (STDs). The overwhelming curricula can lead to cognitive overload and difficulty in retaining critical details [1]. Often, it is difficult to distinguish the vital portions of this information as the clinical correlation ground is not set, disabling students from connecting knowledge to the real world [2].

We noticed a discrepancy in the lack of important knowledge on STDs among the university’s medical students in rotations. The information of neither pre-clinical nor in-rotation students was consistent between themselves, consistent with previous studies [3,4].

This quality improvement project (QIP) aimed to unravel the reasoning behind this inconsistency and lack of information and achieve a significantly higher percentage of students stating they have sufficient and crucial information on STDs by increasing their knowledge via various interventions. The diagnostic goals were to determine preexisting misconceptions and evaluate medical students' baseline knowledge of STDs. Interventional objectives were to increase students' knowledge through educational posters and peer-led instruction and to assess the effects of these interventions over the course of two plan-do-study-act (PDSA) cycles.

## Materials and methods

Design

We employed the PDSA approach in two cycles for our project. To determine the participants' knowledge levels, our team created a 20-item multiple-choice survey sent to the participants voluntarily for a baseline and after each intervention. The study design was prepared with the ‘SMART’ (specific, measurable, achievable, realistic, timely) aim. The chi-square test was performed to compare the proportions of students who selected the correct answer (or misconception) before vs. after the interventions.

To evaluate the effectiveness of the educational interventions, knowledge levels were assessed on a 5-point scale (1=None to 5=Very Good).

Data structure

The dataset was analyzed as independent samples because the student-by-student pairing (ID mapping) was not available in the summary file.

Participants

The participants are students from each year of the Maltepe University Medical School. Stratification was defined a priori by academic year (Years 1-6). While proportional stratified sampling was conceptually planned, participation was voluntary; therefore, final stratum sizes reflected response rates rather than enforced proportional allocation. All academic years were adequately represented.

Overall knowledge score measurement

A weighted composite index was calculated to quantify participant distribution across predefined strata from 1 to 5, 1 being none and 5 being very good in knowledge. The calculation strategy consisted of a weighted summation in which each group size was multiplied by the coefficient of each corresponding number. This approach allows standardized scaling of the index and ensures comparability across different population structures.

$Index ​= (1&times;N1​)+(2&times;N2​)+(3&times;N3​)+(4&times;N4​)+(5&times;N5​)​ / N1+N2+N3+N4+N5$

PDSA first cycle

A 20-item survey was used to determine the participants' baseline knowledge levels upon noticing a discrepancy among students’ levels of knowledge on STDs (Appendix 1). A Mann-Whitney U test was used. This is the most appropriate test for this dataset because the outcome variable (Likert scale 1- 5) is ordinal (ranked categories) rather than strictly continuous, and it does not assume a normal distribution.

Students were given two weeks to fill out the survey. Upon analysis of the results, problems and the underlying causes were established. Based on the results, peer education was chosen as the method of education, as per the request of the participants. Using the information from the first cycle, final-year medical students gave a structured presentation as part of the peer-led educational intervention. During the roughly 45-minute session, common STDs, routes of transmission, prevention strategies, and common misconceptions found in the baseline survey were all thoroughly reviewed, and questions were welcomed at the end of the lecture.

PDSA second cycle

An independent samples t-test was also performed to compare the mean scores, which is a common metric in medical education literature. A second survey was distributed to assess how the presentation had affected participants' knowledge levels and their degree of satisfaction with the intervention at the end of the discussion (Appendix 2). The answering period for the second questionnaire was seven days. The topics needing more improvement were detected based on analysis and enhancing their proficiency was planned to be conducted via posters around the faculty per the request of the participants. Educational posters were put in areas frequented by students. The posters included the key points that remained relatively low based on the survey and required further detailing. Students were able to assess the third survey with the QR code present on the posters to evaluate the efficacy of the second intervention (Appendix 3). The final survey and the posters were open for review for 50 days.

Data collection and analysis

All data were collected between 8 May and 19 July 2024, with each PDSA cycle implemented sequentially. Excel was used for the analysis of the collected data. The knowledge level of each participant was calculated on a scale from 1 to 5, representing their total understanding. The students' baseline knowledge level was determined by calculating the average knowledge score for each survey cycle. After the survey was completed, the same calculation was made again to assess the degree of improvement. This strategy made it possible to evaluate the increase in knowledge over the intervention cycles statistically without being impacted by the number of participants. 

## Results

Baseline knowledge assessment

A weighted composite index was calculated to quantify participant distribution across levels of knowledge. It included a range from 1 to 5, 1 being none and 5 being very good in knowledge. Pre-intervention analysis showed an overall index of 3.56 among 180 students (Table 1).

**Table 1:** Participants’ initial scores regarding level of knowledge

Information level	Score
1 (None)	10
2 (Insufficient)	38
3 (Fair)	108
4 (Good)	360
5 (Very Good)	125
Average	3.56

A total of 180 medical students participated in the initial survey: n=27 (15%) from Year 1, n=27 (15%) from Year 2, n=31 (17.2%) from Year 3, n=29 (16.1%) from Year 4, n=28 (15.5%) from Year 5, and n=38 students (21.1%) from Year 6 (Table 2).

**Table 2:** Distribution of the years of the students who attended the surveys by percentage

Grade	Initial (n=180)	Presentation (n=59)	Poster (n=53)
1	15%	8%	11.32%
2	15%	5%	16.98%
3	17.2%	19%	13.21%
4	16.1%	17%	18.87%
5	15.5%	24%	13.21%
6	21.1%	27%	26.40%

**Figure 1:**
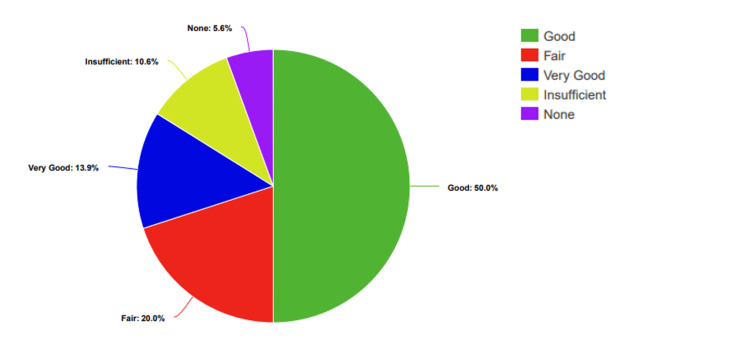
Participants’ percentage-based assessment of knowledge levels regarding STDs STDs: Sexually transmitted diseases

Among the participants, 5.55% (n=10) said they had no knowledge about sexually transmitted infections (STIs), 10.55% (n=19) said they had little knowledge, 20% had a moderate level of knowledge, 50% (n=90) had good knowledge, and 13.88% (n=35) rated their knowledge as very good. Additionally, 79.44% (n=143) of students had already received education about STIs, while 20.55% (n=37) still needed to (Figure 1).

Knowledge levels before and after the presentation

After the presentation, 59 students filled out the survey. The distribution was: n=5 (8%) from Year 1, n=3 (5%) from Year 2, n=11 (19%) from Year 3, n=10 (17%) from Year 4, n=14 (24%) from Year 5, and n=16 (27%) from Year 6 (Table 1).

**Figure 2:**
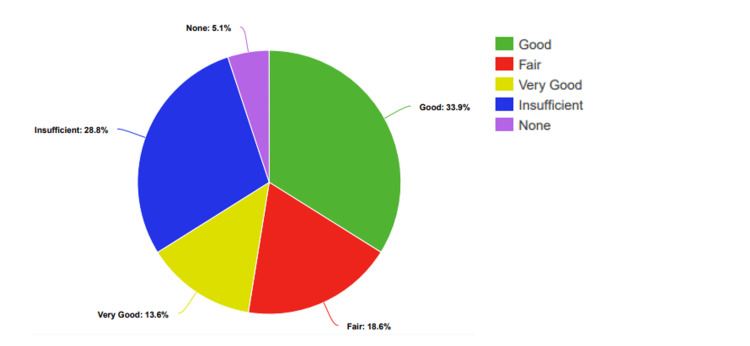
Participants’ percentage-based assessment of knowledge levels regarding STDs, before the presentation STDs: Sexually transmitted diseases

Before the presentation, 5% (n=3) of students rated their knowledge as "None," 29% (n=17) as "Low," 19% (n=11) as "Moderate," 34% (n=20) as "Good," and 14% (n=8) as "Very Good" (Figure 2).

**Figure 3:**
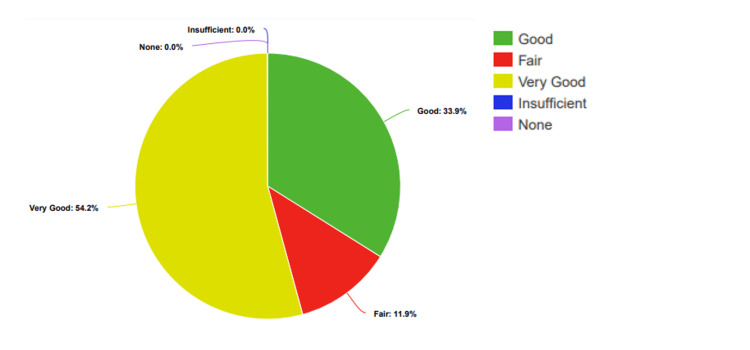
Participants’ percentage-based assessment of knowledge levels regarding STDs, after the presentation STDs: Sexually transmitted diseases

After the presentation, there were clear improvements: no students rated their knowledge as "None" or "Low." Instead, 12% (n=7) rated it as "Fair," 34% (n=20) as "Good," and 54% (n=32) as "Very Good" (Figure 3).

Knowledge levels before and after the poster intervention

After the poster intervention, 53 students completed the survey. The distribution was: n=6 (11.32%) from Year 1, n=9 (6.98%) from Year 2, n=7 (13.21%) from Year 3, n=10 (18.87%) from Year 4, n=7 (13.21%) from Year 5, and n=14 (26.40%) from Year 6 (Table 1).

**Figure 4:**
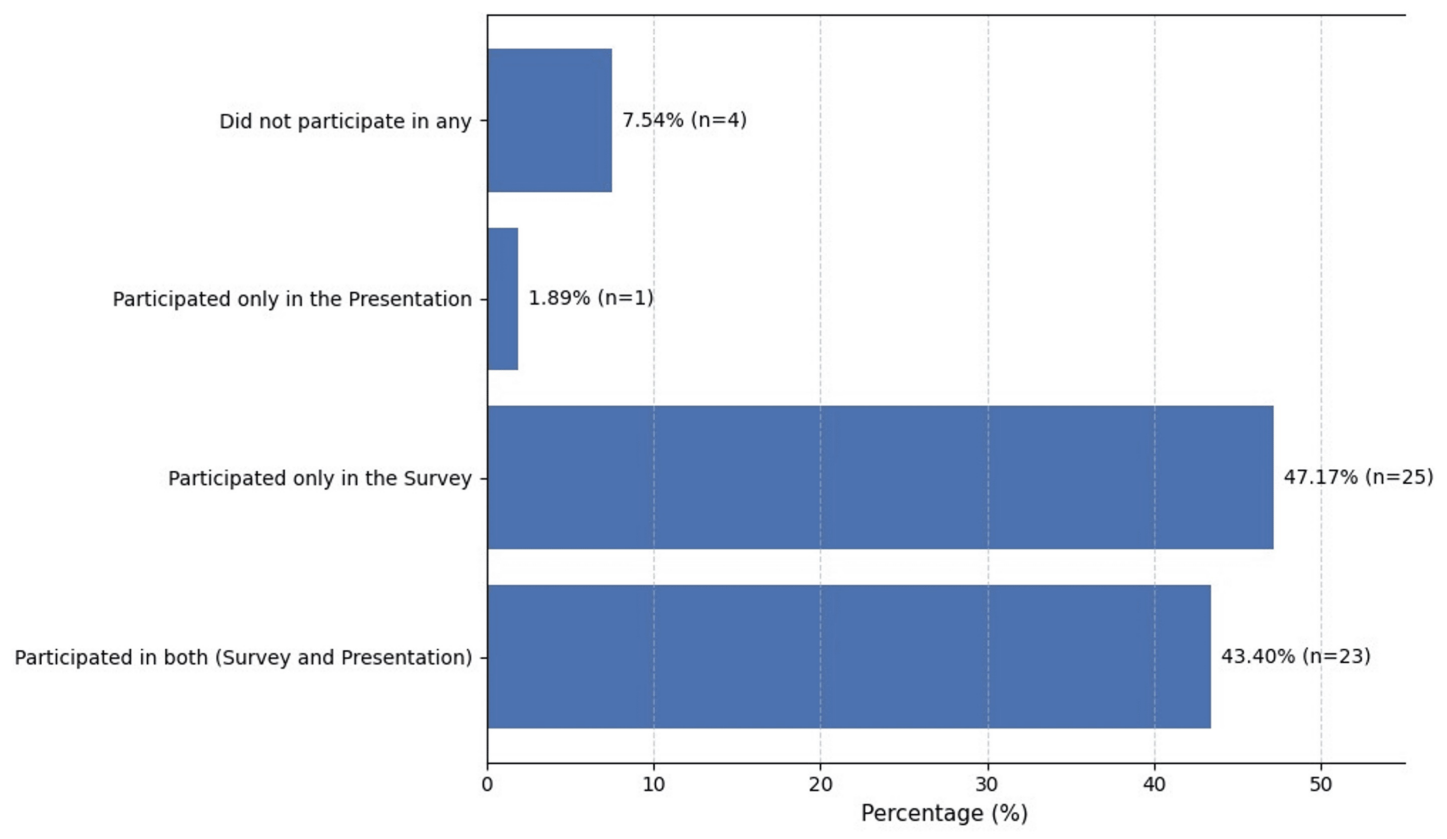
Participants’ response rates to the survey and attendance rates for the presentation

In terms of prior participation, n=25 (47.17%) students had participated in both surveys, n=23 (43.40%) had attended only the survey, and n=1 (1.89%) had attended the presentation (Figure 4).

**Figure 5:**
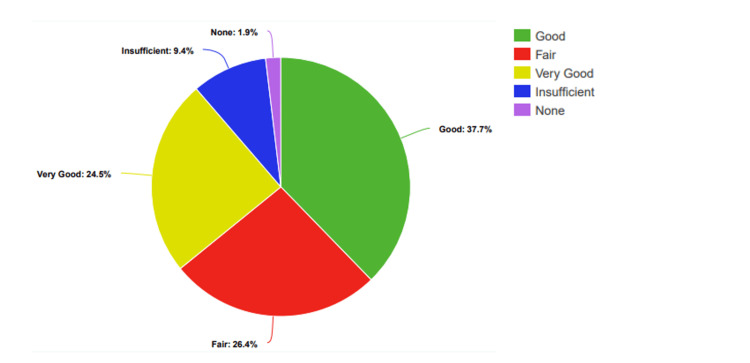
Participants’ percentage-based assessment of knowledge levels regarding STDs, before the poster intervention

Before the posters, n=5 (9.40%) students rated their knowledge as "Low," n=14 (26.40%) as "Fair," n=20 (37.70%) as "Good," and n=13 (24.50%) as "Very Good" (Figure 5).

**Figure 6:**
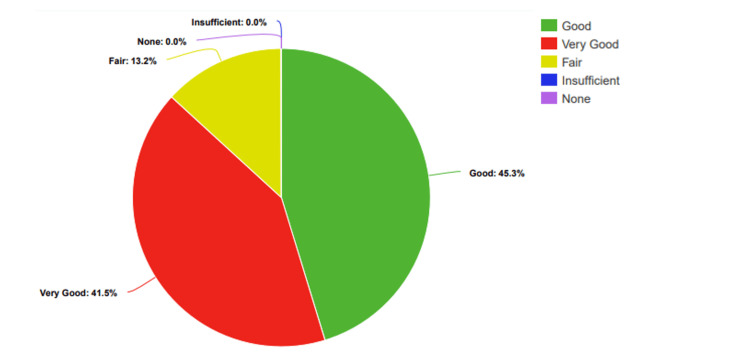
Participants’ percentage-based assessment of knowledge levels regarding STDs, after the poster interventions

After the posters, none of the students rated their knowledge as "Low." Instead, n=7 (13.20%) rated it as "Fair," n=24 (45.30%) as "Good," and n=22 (41.50%) as "Very Good" (Figure 6).

Overall knowledge score improvement

A total knowledge score was computed by assigning numerical values from 1 to 5 for each level of knowledge. In the initial survey of 180 students, the average score was 3.56 (Table 1). For the 59 students in the presentation phase, the mean knowledge score was 3.22 (SD 1.16) and the mean knowledge score post-intervention significantly increased to 4.42 (SD 0.70). The improvement was highly statistically significant (p< 0.001) (Table 3). 

**Table 3:** Mean score improvements after peer and poster education interventions

Intervention	Pre-intervention Mean (SD)	Post-intervention Mean (SD)	p-Value (Mann-Whitney U)	Significance
Peer Education (n=59)	3.22±1.16	4.42±0.70	< 0.001	Significant
Poster Education (n=53)	3.74±1.00	4.28±0.69	0.004	Significant

Among the 53 students in the poster phase, the mean knowledge score was 3.74 (SD 1.00), and the mean knowledge score post-intervention significantly increased to 4.28 (SD 0.69). The improvement was statistically significant (p< 0.004) (Table 3).

Assessment of the helpfulness of the interventions 

Participants were asked about the effectiveness of our interventions in the poster and presentation sessions. The scale was from 1 to 5, 1 representing minimal and 5 representing maximal help. The overall score for the presentation was slightly higher than that for the poster, 4.33 and 3.79, respectively (Table 4). This was asked in order to assess the benefit from the participants’ aspect.

**Table 4:** Participants’ scores on how helpful presentation and poster regarding STDs The scale is from 1 to 5, 1 representing ‘not helpful at all’ and 5 representing ‘very helpful’. STDs: Sexually transmitted diseases

	Presentation Score	Poster Score
1	1	1
2	0	6
3	24	42
4	76	92
5	155	60
Total	4.33	3.79

Knowledge of specific STIs

**Figure 7:**
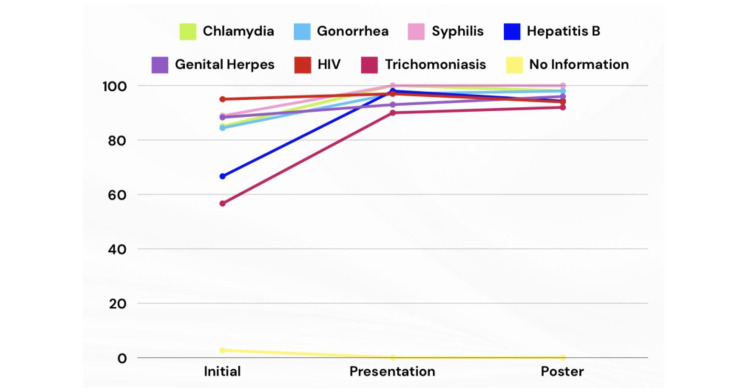
Participants' percentage-based knowledge of specific STDs STDs: Sexually transmitted diseases

In the initial survey, awareness of specific STIs varied: 85% (n=153) were familiar with chlamydia, 84.44% (n=152) with gonorrhea, 88.88% (n=160) with syphilis, 66.66% (n=120) with hepatitis B, 88.33% (n=159) with genital herpes, 95% (n=171) with HIV, and 56.66% (n=102) with trichomoniasis (Figure 7).

Following the presentation, awareness increased for most STIs: 100% (n=59) knew about chlamydia (p = 0.004), 96.6% (n=57) knew about gonorrhea (p < 0.05), 100% (n=59) knew about syphilis (p = 0.016), 98% (n=58) knew about hepatitis B (p < 0.001), 96.6% (n=57) knew about HIV (not significant, p > 0.05), 93.2% (n=55) knew about genital herpes (not significant, p > 0.05), and 90% (n=54) knew about trichomoniasis (p < 0.001).

After the poster intervention, awareness remained high: 98.10% (n=52) knew about chlamydia (p = 0.02), 98.10% (n=52) knew about gonorrhea (p < 0.05), 100% (n=53) knew about syphilis (p = 0.024), 94.30% (n=50) knew about hepatitis B (p < 0.001), 94.30% (n=50) knew about HIV (not significant, p > 0.05), 96.20% (n=51) knew about genital herpes (not significant, p > 0.05), and 92.50% (n=49) knew about trichomoniasis (p < 0.001) (Figure 7).

Perceived transmission routes

**Figure 8:**
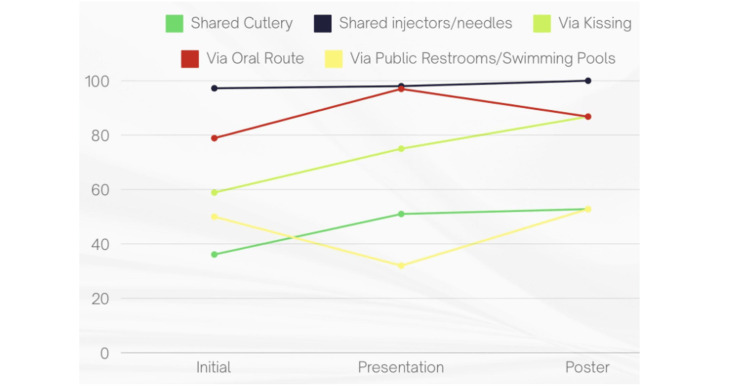
Participants’ percentage-based perceived transmission routes of STDs STDs: Sexually transmitted diseases

In the initial survey, several misconceptions regarding STI transmission were identified among the students. Specifically, 36.11% (n=65) believed that sharing cutlery could spread STIs, 58.88% (n=106) thought kissing could transmit infections, 78.88% (n=142) believed STIs could be transmitted through oral contact, 50% (n=90) thought shared toilets or pools could be a transmission route, and 97.22% (n=175) believed that sharing injection equipment could lead to transmission (Figure 8).

Following the presentation, 51% (n=30) still believed that sharing cutlery could spread STIs (p = 0.06), 75% (n=44) thought kissing was a transmission route (p < 0.001), 97% (n=57) believed in oral transmission, 32% (n=19) still believed STIs could be spread via shared toilets or pools (p = 0.026), and 98% (n=58) thought that sharing injection equipment could transmit infections (Figure 8).

After the display of educational posters, 52.8% (n=28) continued to believe that sharing cutlery could spread STIs (p = 0.84), 86.8% (n=46) thought kissing was a transmission route, 86.8% (n=46) continued to believe in oral transmission, 52.8% (n=28) still believed shared toilets or pools could spread STIs, and 100% (n=53) thought that sharing injection equipment could result in STI transmission (Figure 8).

Knowledge of prevention methods

**Figure 9:**
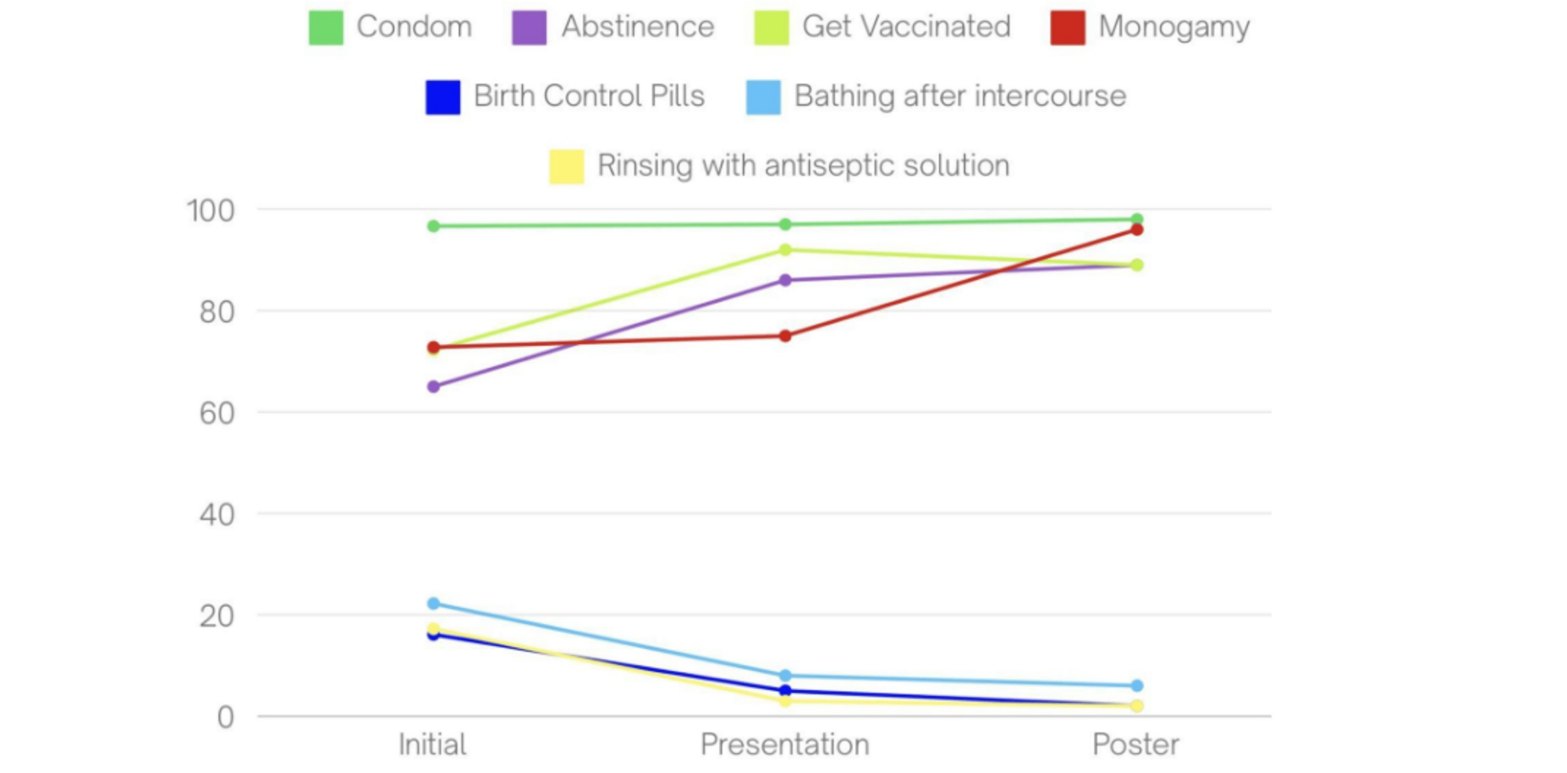
Participants’ percentage-based knowledge of prevention methods of STDs STDs: Sexually transmitted diseases

In the initial survey, the most commonly recognized prevention method was the use of condoms, with 174 students (96.66%) identifying it as protective. Other methods included abstinence, recognized by 117 students (65%), vaccination acknowledged by 72.22%, and monogamy noted by 131 students (72.77%). Fewer students were aware of other methods, such as birth control pills (16.11%), post-intercourse washing (22.22%), and antiseptic baths (17.22%) (Figure 9).

Following the presentation, condom use remained the most recognized prevention method, identified by 97% (n=57), followed by abstinence (n=51, 86%; p < 0.01), vaccination (n=54, 92%; p = 0.004), and monogamy (n=44, 75%). Awareness of other methods decreased, with birth control pills (n=3, 5%), post-intercourse washing (n=5, 8%; p = 0.03), and antiseptic baths (n=2, 3%; p = 0.014) being selected less frequently (Figure 9).

After the poster intervention, condom use remained the most recognized prevention method (n=52, 98.10%), followed by abstinence (n=47, 88.70%; p < 0.01), vaccination (n=47, 89%; p = 0.02), and monogamy (n=44, 96.20%). Other methods continued to show low recognition, with birth control pills (n=1, 1.90%), post-intercourse washing (n=3, 5.70%; p = 0.01), and antiseptic baths (n=1, 1.90%; p = 0.009) (Figure 9).

Misconceptions and testing rights

**Figure 10:**
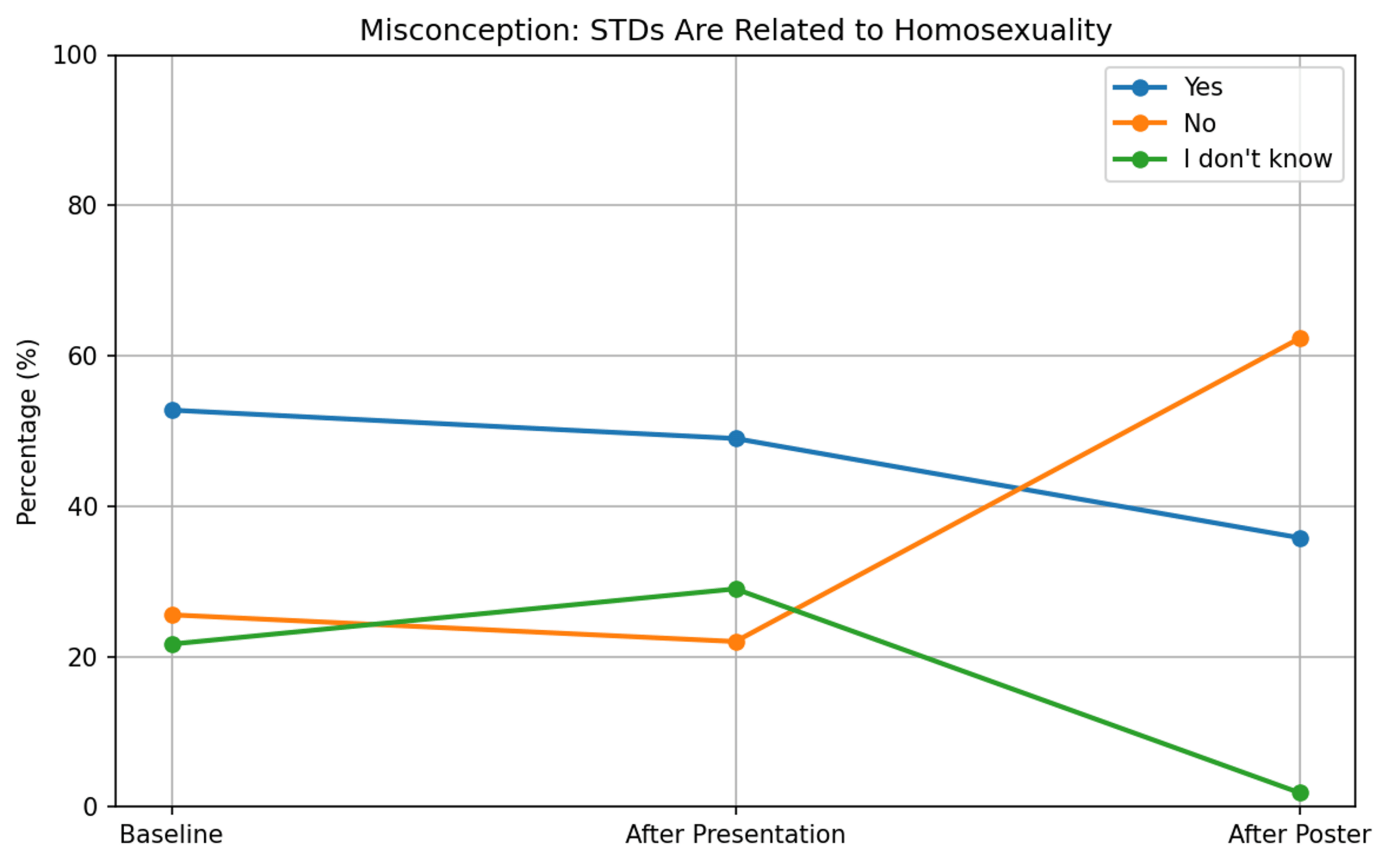
Changes in student misconception that STDs are related to homosexuality across interventions STDs: Sexually transmitted diseases

In the initial survey (n=180), 52.77% (n=95) of students held the opinion that STDs are linked to homosexuality, while 25.55% (n=46) disagreed and 21.66% (n=39) chose "I don't know" (Figure 10).

Following the presentation (n=59), agreement with this misunderstanding fell slightly to 49% (n=29), with no statistically significant difference detected (p=0.74). After the poster intervention (n = 53), the results showed a statistically significant decrease (p = 0.044) in agreement to 35.80% (n = 39), disagreement to 62.30% (n = 33), and uncertainty to 1.90% (n = 1) (Figure 10).

**Figure 11:**
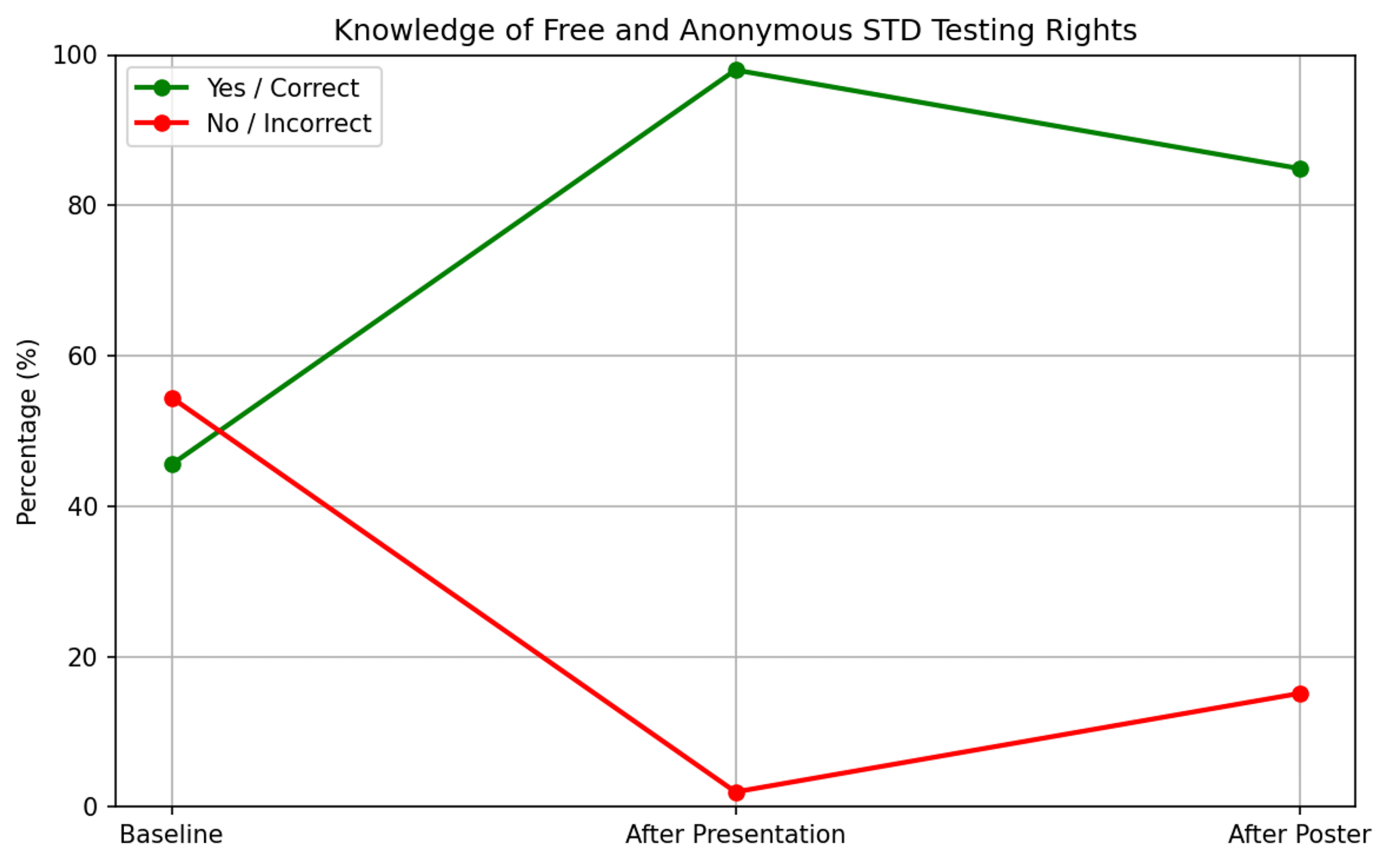
Changes in student knowledge of the right to free and anonymous STD testing STD: Sexually transmitted disease

Knowledge of the right to free and anonymous STD testing was also assessed. 45.55% (n=82) of participants correctly identified this right at baseline, but 54.44% (n=98) did not. Correct answers rose to 98% (n = 58; p < 0.001) after the presentation. Correct identification remained high at 84.90% (n = 45; p < 0.001) following the poster intervention (Figure 11).

## Discussion

The main goal of our QIP was to assess and raise medical students' knowledge of STDs at Maltepe University Faculty of Medicine. The initiative employed peer teaching in the first cycle and educational posters in the second to disseminate knowledge. Improvements in awareness were then measured using surveys after each intervention. The findings were positive, showing overall a considerable increase in awareness across several key areas.

One important measure involved asking participants to rate their level of information regarding STDs on a scale from 1 to 5. This was done both before and after the peer education sessions and poster reviews. This measure was of interest, particularly as it was suggestive of the effectiveness of our intervention. Initially, the average rating was 3.56. It decreased to 3.22 before the peer-education sessions and then rose significantly to 4.42 afterward (p < 0.001). Similarly, after the poster review, the average rating increased from 3.74 to 4.28 (p = 0.004). These changes indicate a significant improvement in participants' knowledge of STDs, underscoring the effectiveness of the interventions.

Additionally, the project’s success is further demonstrated by specific increases in awareness. For example, knowledge of trichomoniasis as an STD increased from 56.66% (n=102) pre-intervention to 92.5% (n=49) post-intervention (p < 0.001), aligning with other studies that emphasize the importance of targeted educational efforts in medical education [5]. Awareness of hepatitis B also rose from 66.66% (n=120) to 94.3% (n=50) (p < 0.001), which is consistent with similar educational campaigns [6]. Moreover, misconceptions, such as the belief that condoms are 100% effective at preventing STDs, were addressed, with 86.8% of participants acknowledging their limitations (p < 0.01).

Another significant outcome was the improvement in students' understanding of the availability of free and anonymous testing, which rose from 45.55% (n=82) to 98% (n=58) after the presentation (p < 0.001) and remained high at 84.9% (n=45) following the poster intervention (p < 0.001). Since we considered this outcome nearly perfect, we shifted our focus to areas that still needed improvement. As a result, the overall percentage decreased in the second cycle, highlighting the need for continuous repetition and exposure. This highlights the importance of comprehensive sexual education, as supported by existing research [7]. The reduction in the belief that STDs are predominantly associated with homosexuality, from 52.77% (n=95) to 35.8% (n=19) (p = 0.044), indicates a more accurate understanding of STD risk factors, consistent with previous studies [8]. 

Finally, the methods of education used in this project were directly influenced by participant feedback. When asked about their preferred modes of education in the final survey, peer education and informative posters emerged as the top two choices. This feedback-driven approach ensured that the interventions were not only effective but also aligned with the preferences and needs of the students.

The choice of peer education and educational posters as our primary intervention methods was guided by several considerations. While other educational approaches such as formal lectures, online modules, or expert-led workshops are widely used in medical education, we opted for methods that were both resource-efficient and aligned with student preferences. Peer education leverages relatability and shared experiences, which can enhance engagement and foster a more open environment for discussing sensitive topics like STDs as demonstrated by a case in Iranian medical students [9]. Similarly, posters offer a passive yet persistent form of information delivery, allowing students to absorb key messages at their own pace. Unlike time-bound lectures or digital modules that require dedicated focus, posters can serve as continuous visual reminders using poster presentation to assess large classes [10]. 

These methods were also selected based on participant feedback and logistical feasibility within the academic setting. Although more structured interventions might yield more uniform results, our chosen methods prioritized accessibility, acceptability, and sustainability, making them particularly suitable for a pilot project conducted within a busy medical school curriculum.

While the results of this project were positive, several limitations must be acknowledged. A major challenge was the inconsistency in student participation, particularly due to the timing of the project, which coincided with final exams. This likely reduced attendance and engagement, thereby limiting the reach and effectiveness of the interventions, which could have inflated post-intervention knowledge scores. For instance, we did not observe a meaningful positive change in the percentages of transmission routes that we attribute to the low participant count. Similar challenges have been noted in other studies, where academic pressures impacted participation in educational programs [11]. 

In addition, the substantial drop in participation, from 180 students in the initial survey to 59 in the peer-education sessions and 53 in the poster review, may have skewed post-intervention knowledge scores toward the positive. Students who remained in later phases may have been more motivated or already more knowledgeable, meaning that the observed improvements could overestimate the true effect across the entire student population. The improvements in mean knowledge scores were highly significant (peer education p < 0.001; poster p = 0.004), but the reduced sample size may exaggerate this effect.

This study stands apart from others in that it was conducted by medical students, for medical students, which facilitated more efficient communication between researchers and participants. However, the lack of a formal authority figure made it difficult to ensure full compliance with all aspects of the intervention. Our experience suggests that future studies could benefit from better scheduling to increase attendance and should incorporate more effective methods to ensure compliance by integrating checkpoints into the curriculum.

Additionally, the possibility that some participants did not respond to surveys with full attention may have introduced bias, potentially affecting the accuracy of the data collected. The absence of a control group represents an important limitation of this study. Lacking the contrast provided by a comparison group, observed knowledge improvements cannot be reliably determined to be due to either the educational interventions themselves or external influences such as other learning that occurred concurrently, growing awareness over time, or repeated exposure to the survey instrument. For these reasons, any causal inferences that might be drawn regarding the effectiveness of the interventions would need to be carefully guarded.

Although the 20-question survey was set up with domains of knowledge for STDs, they have not been formally tested for validity or reliability since its results could potentially have implications for accuracy of measurement. Lastly, since the follow-up period was only relatively short, it is impossible to know if there was actual longer-term knowledge retention or any change in behavior.

## Conclusions

The findings from this project demonstrate that both educational interventions were successful in increasing medical students' understanding of STIs. The positive impact of these interventions is reflected in the overall increase in knowledge scores and the reduction in the number of students with lower knowledge levels. Despite receiving education on related topics, many medical students still held misconceptions about STIs, but when provided with comprehensive and accurate information, they were able to shed these myths. This study is particularly important as it stresses the importance of peer education and teaching by checkpoints. In summary, while the interventions were successful in improving STI knowledge and awareness, addressing issues related to attendance and compliance will be critical for enhancing the impact of similar future projects. To support long-term sustainability, we recommend that peer education sessions and educational materials be embedded into recurring components of the medical school curriculum, such as orientation weeks or public health modules, to ensure continuous exposure and retention of knowledge over time. These findings suggest that student-led educational interventions may be promising, but confirmation through more rigorous and longitudinal study designs is needed.

## Appendices

Appendix 1: pre-seminar survey to assess the students' current level of knowledge

• In order to help us track the data, please write the last four digits of your Turkish ID card or your passport number.

• Which grade are you currently attending?

Grade 1

Grade 2

Grade 3

Grade 4

Grade 5

Grade

• What is your level of information regarding STDs?

Very Good

Good

Fair

Insufficient

None

• If you marked Fair or Insufficient, please explain your reasons regarding the way you think.

(İngilizce yanıtlar:)

There is always more to learn

I feel like I only know the basic medical information, but not in detail

• Have you received an education about STDs previously?

Yes

No

• Which diseases are sexually transmitted diseases (STDs)? (Multiple answers can be selected.)

Chlamydia

Gonorrhea

Syphilis

Hepatitis B

Genital herpes

HIV

Trichomoniasis

I have no information

• If you learn that a colleague of yours is suffering from an STD, would this situation make you have a more avoidance-based attitude?

Yes

No

Not sure

• In your opinion, from the options below, are the routes of transmission of STDs? (Multiple answers can be selected.)

Shared cutlery

Shared injectors or needles

Kissing

Oral route

Public Restrooms or Swimming Pools

• What are the methods of preventing STDs? (Multiple answers can be selected.)

Condom

Abstinence

Get vaccinated

Monogamy

Birth Control Pills

Bathing after intercourse

Rinsing with an antiseptic solution

• Do you think that the use of condoms will protect against all STDs?

Yes

I don't know

No

• Do you think STDs are more related to homosexuality?

Yes

No

I don't have any information related to the subject

• Do you have any information about whether the rate of transmission of STDs from woman to man and from man to woman in heterosexual relationships is the same?

Yes

No

• Can STDs be asymptomatic?

Yes

No

I do not know

• Which STDs are curable? (Multiple answers can be selected.)

Chlamydia

Gonorrhea

HIV

Genital Herpes

Syphilis

Trichomoniasis

• Do you think STDs can cause infertility in women or men?

Yes

No

• Do you know that you have the right to get free and anonymous testing for some STDs?

Yes

No

• Which methods do you think can elevate your knowledge on STDs? (Multiple answers can be selected.)

Increasing lesson hours in the curriculum

Learning via peer education

Reading posters on walls

Joining educational seminars

Appendix 2: post-seminar survey to assess the students' current level of knowledge

 In order to help us track the data, please write the last four digits of your Turkish ID card or your passport number that you used in the first survey.

Which grade are you currently attending?

Grade 1

Grade 2

Grade 3

Grade 4

Grade 5

Grade 6

What was your level of information regarding STDs before the presentation?

Very Good

Good

Fair

Insufficient

None

What is your level of information regarding STDs after the presentation?

Very Good

Good

Fair

Insufficient

None

If you marked Fair or Insufficient, please explain your reasons regarding the way you think. (Open question)

How helpful was our presentation to you? Please rate from 1 to 5.

(1: it was not helpful, 5: it was very helpful)

Have you received an education about STDs prior to the presentation?

Yes

No

Which diseases are sexually transmitted diseases (STDs)? (Multiple answers can be selected.)

Chlamydia

Gonorrhea

Syphilis

Hepatitis B

Genital herpes

HIV

Trichomoniasis

I have no information

If you learn that a colleague of yours is suffering from an STD, would this situation make you have a more avoidance-based attitude?

Yes

No

Not sure

In your opinion, from the options below, are the routes of transmission of STDs? (Multiple answers can be selected.)

Shared cutlery

Shared injectors or needles

Via kissing

Via oral route

Public restrooms or swimming pools

What are the methods of preventing STDs? (Multiple answers can be selected.)

Condom

Abstinence

Get vaccinated

Monogamy

Birth control pills

Bathing after intercourse

Rinsing with an antiseptic solution

Do you think that the use of condoms will protect against all STDs?

Yes

I don’t know

No

Do you think STDs are more related to homosexuality?

Yes

No

I don't have any information related to the subject

Do you have any information about whether the rate of transmission of STDs from woman to man and from man to woman in heterosexual relationships is the same?

Yes

No

Can STDs be asymptomatic?

Yes

No

I do not know

Which STDs are curable? (Multiple answers can be selected.)

Chlamydia

Gonorrhea

HIV

Genital herpes

Syphilis

Trichomoniasis

Do you think STDs can cause infertility in women or men?

Yes

No

Do you know that you have the right to get free and anonymous testing for some STDs?

Yes

No

Appendix 3: post-poster survey to assess the students' current level of knowledge

In order to help us track the data, please write the last four digits of your Turkish ID card or your passport number that you used in the first survey.

Which grade are you currently attending?

Grade 1

Grade 2

Grade 3

Grade 4

Grade 5

Grade 6

Have you participated in our previous survey and the presentation we made about STDs?

I participated in the survey

I attended the presentation

I did not participate in any of them

What is your level of information regarding STDs before viewing the poster?

Very Good

Good

Fair

Insufficient

None

What is your level of information regarding STDs after viewing the poster?

Very Good

Good

Fair

Insufficient

None

If you marked Fair or Insufficient, please explain your reasons regarding the way you think. (Open question)

How helpful was our poster to you? Please rate from 1 to 5.

(1: it was not helpful, 5: it was very helpful)

Have you received any education about STDs before viewing this poster?

Yes

No

Which diseases are sexually transmitted diseases (STDs)? (Multiple answers can be selected.)

Chlamydia

Gonorrhea

Syphilis

Hepatitis B

Genital herpes

HIV

Trichomoniasis

I have no information

If you learn that a colleague of yours is suffering from an STD, would this situation make you have a more avoidance-based attitude?

Yes

No

Not sure

In your opinion, from the options below, are the routes of transmission of STDs? (Multiple answers can be selected.)

Shared cutlery

Shared injectors or needles

Via kissing

Via oral route

Public restrooms or swimming pools

What are the methods of preventing STDs? (Multiple answers can be selected.)

Condom

Abstinence

Get vaccinated

Monogamy

Birth control pills

Bathing after intercourse

Rinsing with an antiseptic solution

Do you think that the use of condoms will protect against all STDs?

Yes

I don’t know

No

Do you think STDs are more related to homosexuality?

Yes

No

I don't have any information related to the subject

Do you have any information about whether the rate of transmission of STDs from woman to man and from man to woman in heterosexual relationships is the same?

Yes

No

Can STDs be asymptomatic?

Yes

No

I do not know

Which STDs are curable? (Multiple answers can be selected.)

Chlamydia

Gonorrhea

HIV

Genital herpes

Syphilis

Trichomoniasis

Do you think STDs can cause infertility in women or men?

Yes

No

Do you know that you have the right to get free and anonymous testing for some STDs?

Yes

No

If you have any ideas about our poster or something that you would like to submit to us, you can write below. (Open question)
